# Supplementary material for: Technology-based group exercise interventions for people living with dementia or mild cognitive impairment: A scoping review
Source: PLoS One. 2024 Jun 13;19(6):e0305266. doi: 10.1371/journal.pone.0305266 (PMC11175425; doi:10.1371/journal.pone.0305266)
Supplement: S1 Appendix — (DOCX) [file pone.0305266.s001.docx]

**Appendix Search strategies example**

**CINAHL database**

| S10 | S3 AND S6 AND S9 | 235 |
| --- | --- | --- |
| S9 | S7 OR S8 | 300,682 |
| S8 | TI ( Exercis* OR physical N1 activit* OR yoga OR tai chi OR taiji OR danc* ) OR AB ( Exercis* OR physical N1 activit* OR yoga OR tai chi OR taiji OR danc* ) | 222,498 |
| S7 | (MH "Exercise+") OR (MH "Physical Activity") | 174,834 |
| S6 | S4 OR S5 | 94,201 |
| S5 | TI ( (internet OR online OR web*) N3 intervent* OR telehealth OR telemedic* OR “mobile health” OR eHealth OR mHealth OR video N1 gam* OR Comput* N1 (gam* OR model* OR simulat*) OR (virtual OR augment*) N1 realit* OR exergam* ) OR AB ( (internet OR online OR web*) N3 intervent* OR telehealth OR telemedic* OR “mobile health” OR eHealth OR mHealth OR video N1 gam* OR Comput* N1 (gam* OR model* OR simulat*) OR (virtual OR augment*) N1 realit* OR exergam* ) | 46,732 |
| S4 | (MH "Internet-Based Intervention") OR (MH "Telehealth+") OR (MH "Computer Simulation+") OR (MH "Video Games+") | 72,242 |
| S3 | S1 OR S2 | 218,225 |
| S2 | TI ( dementia* OR alzheimer* OR cognit* N1 impair* OR memor* N2 los* OR resident* ) OR AB ( dementia* OR alzheimer* OR cognit* N1 impair* OR memor* N2 los* OR resident* ) | 220,882 |
| S1 | (MH "Dementia+") OR (MH "Mild Cognitive Impairment") | 85,906 |
