# Supplementary material for: Technology-based group exercise interventions for people living with dementia or mild cognitive impairment: A scoping review
Source: PLoS One. 2024 Jun 13;19(6):e0305266. doi: 10.1371/journal.pone.0305266 (PMC11175425; doi:10.1371/journal.pone.0305266)
Supplement: Attachment — Submitted filename: Response to Reviewers.docx [file pone.0305266.s003.docx]

Hi Yuchen,

I am writing to submit my revised manuscript entitled “Technology-based group exercise interventions for people living with dementia or mild cognitive impairment: a scoping review” for consideration in the PLOS ONE journal. We sincerely appreciate your valuable comments and suggestions, as they provide our team with a valuable learning opportunity. We have carefully reviewed all the feedback and revised the manuscript accordingly as follows:

When submitting your revision, we need you to address these additional requirements.

1. Please ensure that your manuscript meets PLOS ONE's style requirements, including those for file naming. The PLOS ONE style templates can be found at

https://journals.plos.org/plosone/s/file?id=wjVg/PLOSOne_formatting_sample_main_body.pdf and

<https://journals.plos.org/plosone/s/file?id=ba62/PLOSOne_formatting_sample_title_authors_affiliations.pdf>

- **The manuscript has now been checked to meet style requirements and author affiliation requirements.**

2. Please review your reference list to ensure that it is complete and correct. If you have cited papers that have been retracted, please include the rationale for doing so in the manuscript text, or remove these references and replace them with relevant current references. Any changes to the reference list should be mentioned in the rebuttal letter that accompanies your revised manuscript. If you need to cite a retracted article, indicate the article’s retracted status in the References list and also include a citation and full reference for the retraction notice.

- **The reference list has been reviewed now to ensure its accuracy.**

Additional Editor Comments:
Reviewers' comments:
Reviewer's Responses To Questions

**Comments to the Author**

3. Have the authors made all data underlying the findings in their manuscript fully available?
The [PLOS Data policy](http://www.plosone.org/static/policies.action#sharing) requires authors to make all data underlying the findings described in their manuscript fully available without restriction, with rare exception (please refer to the Data Availability Statement in the manuscript PDF file). The data should be provided as part of the manuscript or its supporting information, or deposited to a public repository. For example, in addition to summary statistics, the data points behind means, medians and variance measures should be available. If there are restrictions on publicly sharing data—e.g. participant privacy or use of data from a third party—those must be specified.

Reviewer #1: Yes

Reviewer #2: No

- **Table 2 has been added to supplement data related to the demographic characteristics of participants.**

5. Review Comments to the Author

Please use the space provided to explain your answers to the questions above. You may also include additional comments for the author, including concerns about dual publication, research ethics, or publication ethics. (Please upload your review as an attachment if it exceeds 20,000 characters)

Reviewer #1: Interesting paper. Well written in good English language. Methodology elaborated well. results and conclusions drawn are appropriate. Discussion is well written. Limitations mentioned as well. Accept without changes

Reviewer #2: The manuscript was well-written. However, the results section provided little raw data to support the textual narrative of the studies. It is a recommendation to add more tables that can synthesize the studies based on the study objectives. The authors are also suggested to provided the strategy done in the synthesis. It was not clear if the results of the data abstraction were synthesized by a sole individual or discussed amongst the group of authors. This could have an implication in the limitations of the study as this can also shape how data is interpreted.

- **The raw data regarding participants’ demographic characteristics has been included in Table 2 to support the textual narrative of the studies now.**
- **Table 1 lists the interventions, outcome measures, feasibility, and results.**
- **The strategies employed, such as identifying key themes and concepts, and comparison and contrast, have been integrated into the discussion section.**
- **Data extraction and synthesis were carried out individually and collectively, as detailed in the methods section now.**
- **The implications brought by this synthesis approach have been addressed in the strengths and limitations section.**

While revising your submission, please upload your figure files to the Preflight Analysis and Conversion Engine (PACE) digital diagnostic tool, <https://pacev2.apexcovantage.com/>. PACE helps ensure that figures meet PLOS requirements. To use PACE, you must first register as a user. Registration is free. Then, login and navigate to the UPLOAD tab, where you will find detailed instructions on how to use the tool. If you encounter any issues or have any questions when using PACE, please email PLOS at [figures@plos.org](mailto:figures@plos.org). Please note that Supporting Information files do not need this step.

- **Fig 1 has been uploaded to PACE to meet PLOS requirements.**

We look forward to the opportunity to contribute to the PLOS ONE journal and are open to any feedback or suggestions for further improvement.

Sincerely,

Lillian Hung
